# Evaluation of stemness properties of cells derived from granulation tissue of peri‐implantitis lesions

**DOI:** 10.1002/cre2.406

**Published:** 2021-02-18

**Authors:** Evangelia Gousopoulou, Athina Bakopoulou, Danae Anastasia Apatzidou, Gabriele Leyhausen, Joachim Volk, Ingmar Staufenbiel, Werner Geurtsen, Knut Adam

**Affiliations:** ^1^ Department of Preventive Dentistry, Periodontology & Implant Biology, School of Dentistry, Faculty of Health Sciences Aristotle University of Thessaloniki (AUTh) Thessaloniki Greece; ^2^ Department of Conservative Dentistry, Periodontology & Preventive Dentistry, School of Dentistry Hannover Medical School (MHH) Hannover Germany; ^3^ Department of Prosthodontics, School of Dentistry, Faculty of Health Sciences Aristotle University of Thessaloniki (AUTh) Thessaloniki Greece

**Keywords:** angiogenic differentiation, inflammatory granulation tissue, mesenchymal stromal cells, neurogenic differentiation, osteogenic differentiation, peri‐implantitis

## Abstract

**Objectives:**

Peri‐implantitis (PI) is an inflammatory disease associated with peri‐implant bone loss and impaired healing potential. There is limited evidence about the presence of mesenchymal stromal cells (MSCs) and their regenerative properties within the granulation tissue (GT) of infrabony peri‐implantitis defects. The aim of the present study was to characterize the cells derived from the GT of infrabony PI lesions (peri‐implantitis derived mesenchymal stromal cells—PIMSCs).

**Material and Methods:**

PIMSC cultures were established from GT harvested from PI lesions with a pocket probing depth ≥6 mm, bleeding on probing/suppuration, and radiographic evidence of an infrabony component from four systemically healthy individuals. Cultures were analyzed for embryonic (SSEA4, NANOG, SOX2, OCT4A), mesenchymal (CD90, CD73, CD105, CD146, STRO1) and hematopoietic (CD34, CD45) stem cell markers using flow cytometry. PIMSC cultures were induced for neurogenic, angiogenic and osteogenic differentiation by respective media. Cultures were analyzed for morphological changes and mineralization potential (Alizarin Red S method). Gene expression of neurogenic (NEFL, NCAM1, TUBB3, ENO2), angiogenic (VEGFR1, VEGFR2, PECAM1) and osteogenic (ALPL, BGLAP, BMP2, RUNX2) markers was determined by quantitative RT‐PCR.

**Results:**

PIMSC cultures demonstrated high expression of embryonic and mesenchymal stem cell markers with inter‐individual variability. After exposure to neurogenic, angiogenic and osteogenic conditions, PIMSCs showed pronounced tri‐lineage differentiation potential, as evidenced by their morphology and expression of respective markers. High mineralization potential was observed.

**Conclusions:**

This study provides evidence that MSC‐like populations reside within the GT of PI lesions and exhibit a multilineage differentiation potential. Further studies are needed to specify the biological role of these cells in the healing processes of inflamed PI tissues and to provide indications for their potential use in regenerative therapies.

## 1 INTRODUCTION

Peri‐implantitis (PI) is a biofilm‐associated inflammatory disease resulting in progressive loss of supporting bone (Berglundh et al., 2018). Up to date there is no predictable therapy for PI. Due to implant surface characteristics and limited access to the microbial habitat, non‐surgical therapy is frequently inefficient in ceasing the progression of the disease. Therefore, surgical intervention is required more often and at an earlier stage in PI lesions, when compared to periodontitis lesions (Heitz‐Mayfield & Lang, 2010).

**FIGURE 1:**
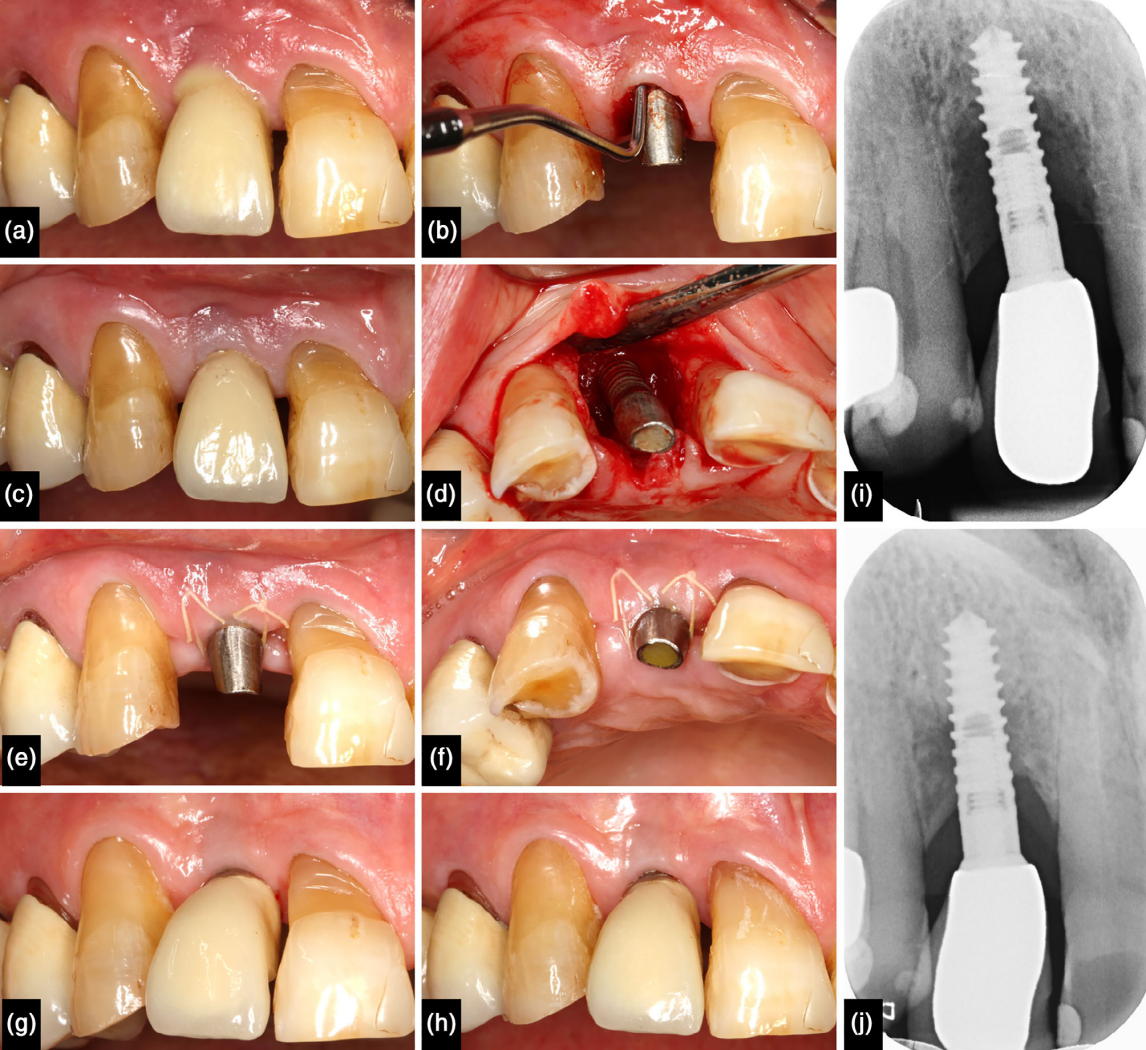
Non‐surgical and surgical treatment of peri‐implantitis. (a) Purulent peri‐implantitis lesion at implant regio 12. (b) Clinical view after removal of the cemented crown. Note the increased probing pocket depth at the buccal aspect of the implant. (c) Clinical situation 2 weeks after non‐surgical treatment using an air‐polishing device. Note the resolution of inflammation signs. (d) After mobilization of the mucoperiosteal flap, a circumferential infrabony defect was detected. The surgical treatment included decontamination of the implant surface and application of enamel matrix derivatives. (e and f) Apart from a minor soft tissue dehiscence at the mesial papilla, an uneventful wound healing was observed 1 week after surgery. (g and h) Bland mucosal conditions 1 and 2 years after surgery without formation of mucosal recession. (i and j) A pronounced infrabony component was present on the baseline X‐ray. A significant bone fill was achieved 2 years after surgery

Similar to periodontitis, PI lesions are dominated by plasma cells and lymphocytes, but with larger shares of polymorphonuclear leukocytes and macrophages (Berglundh et al., 2004, 2011; Bullon et al., 2004; Cornelini et al., 2001; Sanz et al., 1991). Besides, it was documented that the size of PI lesions is more than twice as large as that noted at periodontitis sites (Carcuac & Berglundh, 2014). Moreover, PI lesions are characterized by a higher density of vascular structures lateral to the infiltrated connective tissue (Carcuac & Berglundh, 2014). Recently, we observed that granulation tissue (GT) harvested from periodontal pockets exhibit a cellular infiltrate reflecting a chronic inflammatory state (Apatzidou et al., 2018). Due to the presence of mesenchymal stromal cells (MSCs) and immunophenotypic characteristics similar to those found in clinically healthy periodontal tissues, it can be concluded that periodontal GT, albeit inflamed, retain regenerative healing potential (Apatzidou et al., 2018). For both, infrabony periodontitis and PI lesions, we could show that the preservation of GT during regenerative surgery may improve the treatment outcome from a clinical and radiographic perspective. Thus, application of the granulation tissue preservation technique (GTPT) resulted in significant clinical attachment gain and radiographic bone fill (Figure 1; Günay et al., 2013, 2019).

The presence of mesenchymal stromal cells in the GT derived from infrabony PI defects (peri‐implantitis derived mesenchymal stromal cells = PIMSC) has not been investigated so far. Therefore, the aim of the present study was to investigate whether cell cultures established from the GT of PI lesions show stemness properties, including expression of mesenchymal and embryonic stem cell markers and multipotent differentiation potential. Donos et al. investigated the gene expression profile following placement of dental implants in humans (Donos, Hamlet, et al., 2011; Donos, Retzepi, et al., 2011). They demonstrated that the biological processes of osteogenesis, angiogenesis and neurogenesis play a fundamental role in osseointegration. Although scientific evidence is lacking so far, it seems likely that the same biological processes are required for the regeneration of infrabony PI defects. Therefore, the current study selected the neurogenic, angiogenic and osteogenic pathways to show multipotency.

## 2 MATERIALS AND METHODS

### 2.1 Establishment of PIMSC cultures

The human PIMSC cultures used in the present study were established from PI lesions of four male donors aged 52–68 years. All donors were systemically healthy, not taking any medication and not consuming any alcohol. Two of the donors were smokers with a daily consumption of more than 15 cigarettes; the other two donors were non‐smokers. All patients received non‐surgical therapy to reduce local signs of inflammation and to facilitate the surgical intervention. Before surgery, a residual PI lesion with a pocket probing depth ≥6 mm, bleeding on probing, and a radiographically evident infrabony defect had to be present (Renvert et al., 2018). After mobilization of the muco‐periosteal flap, the inflammatory GT was collected from the defect using curettes and scalers. In particular the GT from the bottom of the PI lesion was harvested. Cell cultures were established using the enzymatic dissociation method, as described previously (Bakopoulou et al., 2010). Briefly, the tissue samples were cut into small pieces and digested in alpha‐minimal essential medium (alpha‐MEM, Gibco, Grand Island, NY) supplemented with 3 mg/mL collagenase type I (Gibco/Life Technologies, Paisley, Scotland) and 4 mg/mL dispase II (Sigma‐Aldrich, Steinheim, Germany) for 1 h at 37°C. Filtration through a strainer with a pore size of 70 μm (EASYstrainer, Greiner bio‐one, Frickenhausen, Germany) eliminated tissue debris. The resulting single‐cell suspension was seeded into cell culture flasks containing complete culture medium (CCM). This consisted of alpha‐MEM supplemented with 15% fetal bovine serum (FBS, Biochrom, Berlin, Germany), 100 U/mL penicillin (Biochrom), 100 μg/mL streptomycin (Biochrom), 2.5 μg/mL amphotericin B (Capricorn Scientific, Ebsdorfergrund, Germany), and 100 μM L‐ascorbic acid phosphate (Sigma‐Aldrich). The cells were incubated in humidified atmosphere at 37°C in 5% CO_2_ and the first medium change was carried out after 24 h to remove the abundance of erythrocytes. After reaching 80%–90% confluency, cells were collected by treatment with a 0.25% trypsin/EDTA solution (Gibco) and continuously passaged for further experiments. PISMC cultures of the passages 2–5 were used for all experiments with similar results. The present study has been approved by the Institutional Review Board (Ethics Committee of Hannover Medical School, reference number: 1096) and all donors signed an informed consent according to the Declaration of Helsinki.

### 2.2 Characterization of PIMSC cultures with flow cytometry

The antigen profiles of PIMSC cultures were analyzed by flow cytometry. Cells were seeded in 75 cm^2^ culture flasks and expanded in CCM until confluency. Cells were detached from the culture vessels by treatment with 0.25% trypsin/EDTA, washed with phosphate‐buffered saline (PBS), and re‐suspended in FACS buffer consisting of PBS supplemented with 1% bovine serum albumin (BSA) and 0.1% sodium azide (NaN_3_). For each sample, 1 × 10^6^ cells/100 μL FACS buffer were Fc‐blocked with 1 μg of human IgG (Sigma‐Aldrich) for 15 min on ice. The staining was carried out by incubation with the following fluorochrome‐conjugated mouse anti‐human antibodies for 25 min in the dark on ice: CD90‐FITC (fluorescein isothiocyanate), CD73‐FITC, CD105‐APC (allophycocyanin), CD146‐PE (phycoerythrin), STRO1‐FITC, SSEA4‐FITC, CD34‐APC, and CD45‐PE (all from BioLegend, Fell, Germany). For intracellular staining, cells were fixed with 4% paraformaldehyde buffer (BD Biosciences, Heidelberg, Germany), permeabilized with 0.1% saponin buffer (BD Biosciences), and subsequently stained by incubation with the mouse anti‐human antibodies NANOG‐PE, OCT4A‐Alexa Fluor 647 and SOX2‐PE (all from BD Biosciences) for 25 min on ice in the dark. For flow cytometry analyses, a BD LSR II Flow Cytometer (BD Biosciences) was used. A total of 100,000 events were acquired for each sample. Processing of the raw data was performed using BD FACSDiva™ Software (BD Biosciences) and Summit 5.1 software (Beckman Coulter, Inc., Fullerton, CA).

### 2.3 Neurogenic differentiation

For the induction of neurogenic differentiation, PIMSCs were seeded into six‐well plates coated with 0.1% gelatin (Sigma‐Aldrich) at 1 × 10^5^ cells / well and grown in neurogenic differentiation medium (NDM). This consisted of neurobasal A medium (Gibco) supplemented with B27 supplement (2% v/v, Gibco), 2 mM L‐glutamine (Gibco), 20 ng/mL recombinant human epidermal growth factor (rh‐EGF, Biochrom), 40 ng/mL recombinant human basic fibroblast growth factor (rh‐bFGF, Biochrom), 100 U/mL penicillin, 100 μg/mL streptomycin, and 2.5 μg/mL amphotericin B. Cells were cultured for 5 weeks and the NDM was changed every 2–3 days. Cultures exposed to CCM were used as negative control. Neurogenic differentiation was assessed by observation under an inverted microscope for the detection of morphological changes towards a neuron‐like phenotype and by real‐time reverse transcriptase polymerase chain reaction (qRT‐PCR) for the expression of neural markers, including neurofilament light polypeptide (NEFL), neural cell adhesion molecule 1 (NCAM1), tubulin beta 3 class III (TUBB3) and enolase 2 (ENO2).

### 2.4 Angiogenic differentiation

For the induction of angiogenic differentiation, PIMSCs were seeded into six‐well plates coated with collagen I (Santa Cruz Biotechnology, Heidelberg, Germany) at 1 × 10^5^ cells / well and expanded in CCM until they reached confluency. Afterwards, cells were exposed to angiogenic differentiation medium (ADM) consisting of M199 medium (Gibco) supplemented with 5% FBS, 100 U/mL penicillin, 100 μg/mL streptomycin, 2.5 μg/mL amphotericin B, 50 μg/mL heparin (Sigma‐Aldrich), 1 μg/mL hydrocortisone (Sigma‐Aldrich), 60 μg/mL endothelial cell growth supplement (ECGS, PromoCell, Heidelberg, Germany), 10 ng/mL rh‐EGF, 25 ng/mL rh‐bFGF, and 50 ng/mL recombinant human vascular endothelial growth factor (rh‐VEGF, Gibco). Cells were cultured for 5 weeks and the ADM was changed every 2–3 days. Angiogenic differentiation was assessed by morphological characteristics and qRT‐PCR for the expression of angiogenic markers, including vascular endothelial growth factor receptor 1 (VEGFR1), vascular endothelial growth factor receptor 2 (VEGFR2), and platelet and endothelial cell adhesion molecule 1 (PECAM1).

### 2.5 Osteogenic differentiation

For the induction of osteogenic differentiation, PIMSCs were seeded into six‐well plates at 1 × 10^5^ cells / well and expanded in CCM until they reached confluency. Subsequently, cells were exposed to osteogenic differentiation medium (ODM) consisting of CCM supplemented with 5 mM β‐glycerol phosphate (Sigma‐Aldrich), 1.8 mM monopotassium phosphate (KH_2_PO_4_, Sigma‐Aldrich), and 10 nM dexamethasone (Sigma‐Aldrich). Cells were cultured for 4 weeks and the ODM was changed every 2–3 days. Osteogenic differentiation was analyzed using the Alizarin Red S (AR‐S) method to identify the mineralized matrix. Briefly, the culture dishes were washed two times with PBS without Ca^2+^ and Mg^2+^ (Biochrom), and cells were fixed with 10% (w/v) neutral buffered formalin solution (Sigma‐Aldrich) for 1 h at room temperature (RT). Afterwards, the culture dishes were washed two times with distilled water and staining was performed using 1% AR‐S (pH 4.0, Sigma‐Aldrich) for 20 min at RT. The culture dishes were washed four times with 2 mL distilled water to eliminate unspecific staining and mineralized deposits were visualized and photographed under an inverted microscope equipped with a digital camera (Olympus Optical Co., Ltd., Japan). Quantification of mineralized matrix was performed by the AR‐S extraction method. After aspiration of the distilled water, 1.5 mL cetylpyridinium chloride buffer (CPC, 10%, w/v, dissolved in 10 mM disodium monohydrogen phosphate, pH 7) was added to the culture dishes for 2 h at 37°C. Aliquots of 200 μL were transferred to a 96‐well plate. A microplate spectrophotometer was used to measure the optical absorption at 550 nm (Spectra Max 250, MWG Biotech, Sunnyvale, CA). In addition, the qRT‐PCR was applied to evaluate the expression of osteogenic markers, including runt‐related transcription factor 2 (RUNX2), bone gamma‐carboxyglutamate protein or osteocalcin (BGLAP), bone morphogenic protein 2 (BMP2) and alkaline phosphatase (ALPL).

### 2.6 Quantitative real‐time reverse transcription polymerase chain reaction

A two‐step quantitative real‐time reverse transcription polymerase chain reaction (qRT‐PCR) was applied to analyze changes in gene expression during neurogenic, angiogenic and osteogenic differentiation. The entire RNA was isolated from the cell cultures using the RNeasy Plant Mini Kit (Qiagen, Hilden, Germany). The genomic DNA was eliminated through on‐column digestion (RNase‐free DNase Set, Qiagen). A microplate reader (Synergy H1, BioTek, Bad Friedrichshall, Germany) was used for the measurement of the RNA concentration. The cDNA was synthesized using 1 μg of isolated RNA and the QuantiTect Reverse Transcription Kit (Qiagen). The QuantiTect SYBR Green PCR Kit, the QuantiTect Primer Assays (Table 1) and the Rotor‐Gene Q cycler (all from Qiagen) were used for the amplification and real‐time quantification of cDNA targets. The PCR reactions involved an initial activation of the HotStarTaq DNA polymerase (at 95°C for 5 min) and 40 cycles of denaturation (at 95°C for 5 s), annealing and extension (at 60°C for 10 s). The specificity of the reaction products was confirmed by a standard melting curve. LinRegPCR was applied to perform baseline correction, to determine the window‐of‐linearity, and to analyze the PCR efficiency per sample and per group (Ruijter et al., 2009). Actin beta (ACTB), beta‐2‐microglobulin (B2M), glyceraldehyde‐3‐phosphate dehydrogenase (GAPDH), 18S ribosomal RNA (RRN18S), succinate dehydrogenase flavoprotein subunit (SDHA2), and tyrosine 3‐monooxygenase/tryptophan 5‐monooxygenase activation protein zeta (YWHAZ) were used as housekeeping genes. The two most stable housekeeping genes were selected by geNorm and used to normalize the adjusted qPCR data (Vandesompele et al., 2002). The delta delta CT method was applied to calculate fold changes in gene expression (Pfaffl, 2001).

**TABLE 1:** QuantiTect Primer Assays (Qiagen) used for the qRT‐PCR analyses

	QuantiTect Primer Assay	Protein / enzyme (abbreviation)	Catalogue number	Detected transcript(s)
**Neurogenic**	Hs_ENO2_1_SG	Enolase 2 (ENO2)	QT00084889	NM_001975 (2423 bp)
	Hs_NCAM1_1_SG	Neural cell adhesion molecule (NCAM1)	QT00071211	NM_000615 (5977 bp) NM_001076682 (4944 bp) NM_001242608 (4831 bp)
	Hs_NEFL_1_SG	Neurofilament, light polypeptide (NEFL)	QT00096369	NM_006158 (3854 bp)
	Hs_TUBB3_1_SG	Tubulin, beta 3 class III (TUBB3)	QT00083713	NM_006086 (1794 bp)
**Angiogenic**	Hs_PECAM1_1_SG	Platelet and endothelial cell adhesion molecule 1 (PECAM1)	QT00081172	NM_000442 (6831 bp) XM_005276880 (4006 bp) XM_005276881 (3972 bp) XM_005276882 (3966 bp) XM_005276883 (3943 bp) XM_006721944 (2438 bp) XM_006721945 (2452 bp)
	Hs_FLT1_1_SG	Fms‐related tyrosine kinase 1 (FLT1) or vascular endothelial growth factor receptor 1 (VEGFR1)	QT00073640	NM_002019 (7123 bp)
	Hs_KDR_1_SG	Kinase insert domain receptor (KDR) or vascular endothelial growth factor receptor 2 (VEGFR2)	QT00069818	NM_002253 (6055 bp)
**Osteogenic**	Hs_ALPL_1_SG	Alkaline phosphatase (ALPL)	QT00012957	NM_000478 (2606 bp) NM_001127501 (2441 bp) NM_001177520 (2325 bp) XM_005245818 (2573 bp) XM_005245820 (1379 bp) XM_006710546 (2558 bp)
	Hs_BGLAP_1_SG	Bone gamma carboxyglutamic acid‐containing protein (BGLAP)	QT00232771	NM_199173 (562 bp)
	Hs_BMP2_1_SG	Bone morphogenic protein 2 (BMP2)	QT00012544	NM_001200 (3150 bp)
	Hs_RUNX2_1_SG	Runt‐related transcription factor 2 (RUNX2)	QT00020517	NM_001015051 (5487 bp) NM_001024630 (5553 bp) NM_004348 (5720 bp) NM_001278478 (5235 bp) XM_006715231 (5304 bp) XM_006715233 (2944 bp) XM_006715234 (875 bp)
**Housekeeping genes**	Hs_RRN18S_1_SG	18S ribosomal RNA (RRN18S)	QT00199367	X03205 (1869 bp)
	Hs_ACTB_1_SG	Actin, beta (ACTB)	QT00095431	NM_001101 (1852 bp)
	Hs_B2M_1_SG	Beta‐2‐microglobulin (B2M)	QT00088935	NM_004048 (987 bp) XM_005254549 (424 bp) XM_006725182 (424 bp)
	Hs_GAPDH_2_SG	Glyceraldehyde‐3‐phosphate dehydrogenase (GAPDH)	QT01192646	NM_002046 (1421 bp) NM_001289745 (1513 bp)
	Hs_SDHA_2_SG	Succinate dehydrogenase complex flavoprotein subunit A (SDHA2)	QT01668919	NM_004168 (2803 bp) NM_001294332 (2659 bp) XM_005248329 (2245 bp) XM_005248331 (2151 bp)
	Hs_YWHAZ_2_SG	Tyrosine 3‐monooxygenase/tryptophan 5‐monooxygenase activation protein, zeta (YWHAZ)	QT02321522	NM_001135699 (3020 bp) NM_001135700 (2974 bp) NM_001135701 (3023 bp) NM_001135702 (3042 bp) NM_003406 (3003 bp) NM_145690 (3077 bp) XM_005251060 (3295 bp) XM_005251061 (3390 bp) XM_005251062 (3165 bp) XM_005251063 (3152 bp)

### 2.7 Statistical analysis

All assays were performed with cell cultures of all four donors. There was a significant inter‐individual biological variability with regard to the qRT‐PCR results. Therefore, the standardization method described by Willems et al. was applied (Willems et al., 2008). This method consisted of a logarithmic transformation, mean centering and autoscaling. The results were expressed as mean values (MV) ± standard deviations (*SD*). One‐way analysis of variance (ANOVA) with Dunnett's multiple comparison test was used to identify significant differences in gene expression compared to the baseline value recorded at day 0.

One‐way ANOVA with Bonferroni's multiple comparison test was applied to detect statistically significant differences in matrix mineralization between cell cultures grown in CCM and ODM, respectively. All analyses were performed using GraphPad Prism 6.0 for Windows (GraphPad, La Jolla, CA). The level of statistical difference was 0.05 (*p* ≤ 0.05).

## 3 RESULTS

### 3.1 Morphological characteristics of PIMSC cultures

**FIGURE 2:**
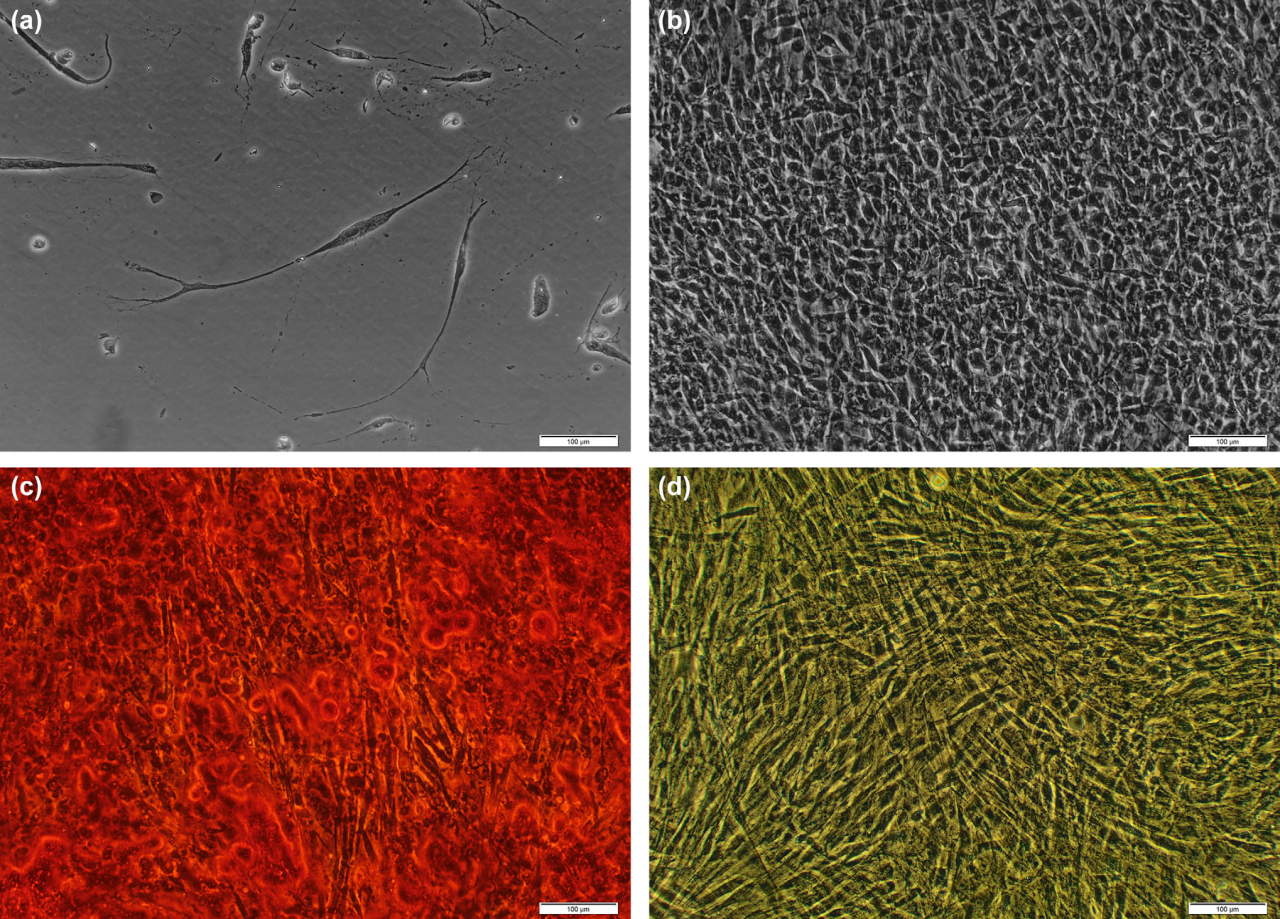
Morphological changes during culture in (a) neurogenic, (b) angiogenic, (c) osteogenic differentiation medium, and (d) complete culture medium. (a) Note the formation of axon‐ and dendrite‐like cell structures after 28 days of induction with neurogenic differentiation medium. (b) Polygonal cells were arranged in a cobblestone‐like organizational pattern after 35 days of induction with angiogenic differentiation medium. (c) An extensive matrix mineralization positively stained by Alizarin Red S was observed after 21 days of culture in osteogenic differentiation medium. (d) Negligible staining was present after 21 days of culture in complete culture medium

Significant morphological differences were observed in PIMSC cultures after stimulation with the corresponding inductive media (Figure 2). Neurogenic differentiation was demonstrated as early as day 3 by morphological changes from fibroblast‐like to neuron‐like cells with a long and slim cell body and extended dendrite‐like branches. The neuron‐like cells were arranged in waves crossing at different sites. In ADM, differentiated cells exhibited a cobblestone‐like organizational pattern and changed from a spindle‐shaped to a more polygonal cellular morphology. In cell cultures grown in ODM, the matrix mineralization started between day 14 and 21 and finally by day 28 the mineralized tissue formation covered the whole surface of the well.

### 3.2 Immunophenotypic characterization

**FIGURE 3:**
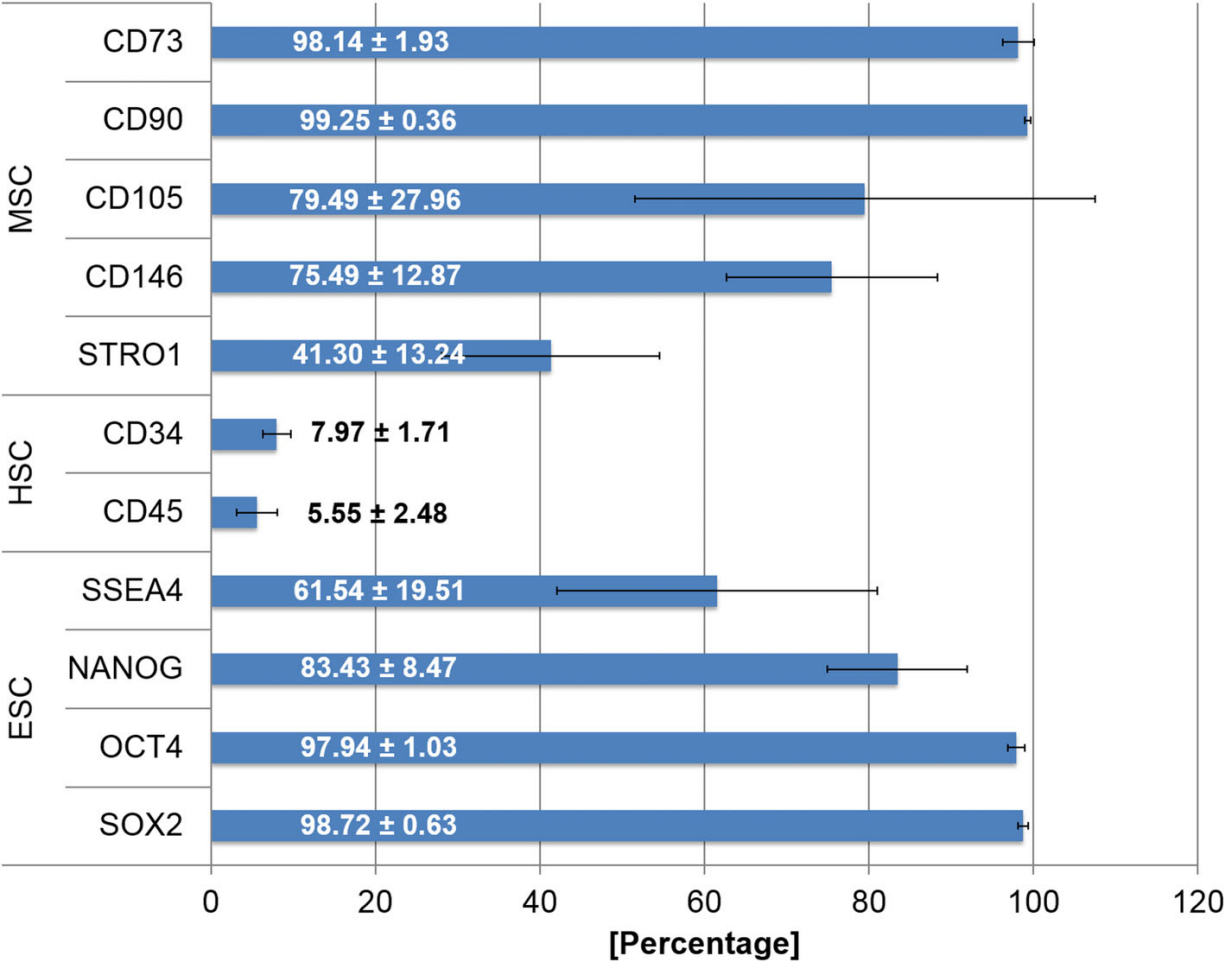
Immunophenotypic characterization of PIMSC cultures using flow cytometry. Expression of mesenchymal (MSC: CD73, CD90, CD105, CD146, STRO1), hematopoietic (HSC: CD34, CD45) and embryonic (ESC: SSEA4, NANOG, OCT4, SOX2) stem cell markers. The summarized data of all donors (*n* = 4) are presented as mean value ± standard deviation

**FIGURE 4:**
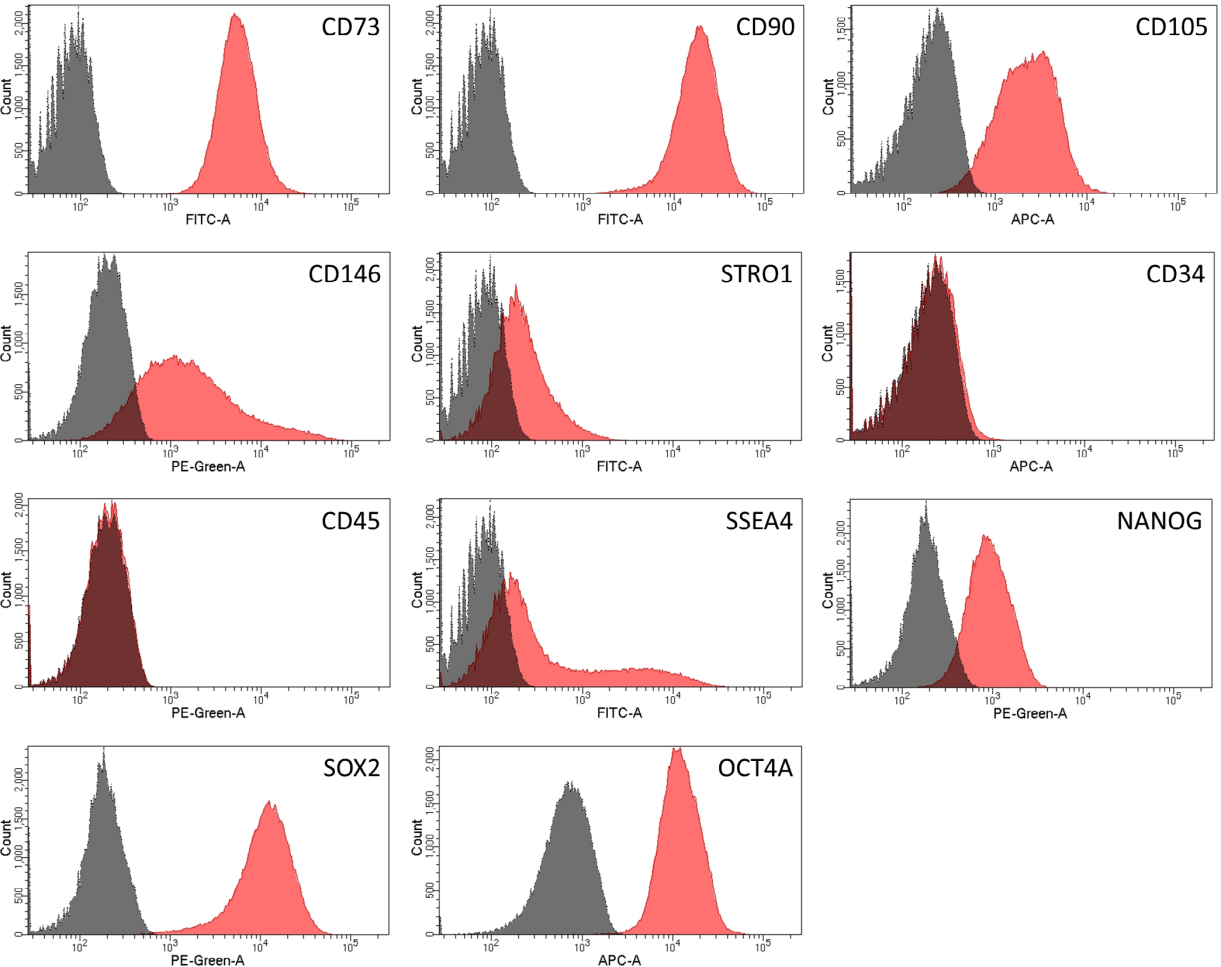
Representative flow cytometry histograms. Expression of mesenchymal (CD73, CD90, CD105, CD146, STRO1), hematopoietic (CD34, CD45) and embryonic (SSEA4, NANOG, OCT4, SOX2) stem cell markers. Dark gray: unstained control. Red: marker of interest

The PIMSC cultures used in this study were highly positive (>98% of the population) for the MSC markers CD90 and CD73. Lower expression was observed for the MSC markers CD146 (75.49% ± 12.87%) and STRO1 (41.30% ± 13.24%). The endothelial stem cell marker CD105 (79.49% ± 27.96%) was also highly expressed. A lower expression was observed for the hematopoietic marker CD34 (7.97% ± 1.71%). The leucocyte precursor marker CD45 (5.55% ± 2.48%) was slightly expressed. Finally, among the various embryonic stem cell markers tested, high expression was observed for SOX2 (98.72% ± 0.63%), OCT4A (97.94% ± 1.03%), SSEA4 (61.54% ± 19.51%) and NANOG (83.43% ± 8.47%). The results of the immunophenotypic analysis are shown in Figures 3 and 4.

### 3.3 Time‐course gene expression of neurogenesis‐related markers

**FIGURE 5:**
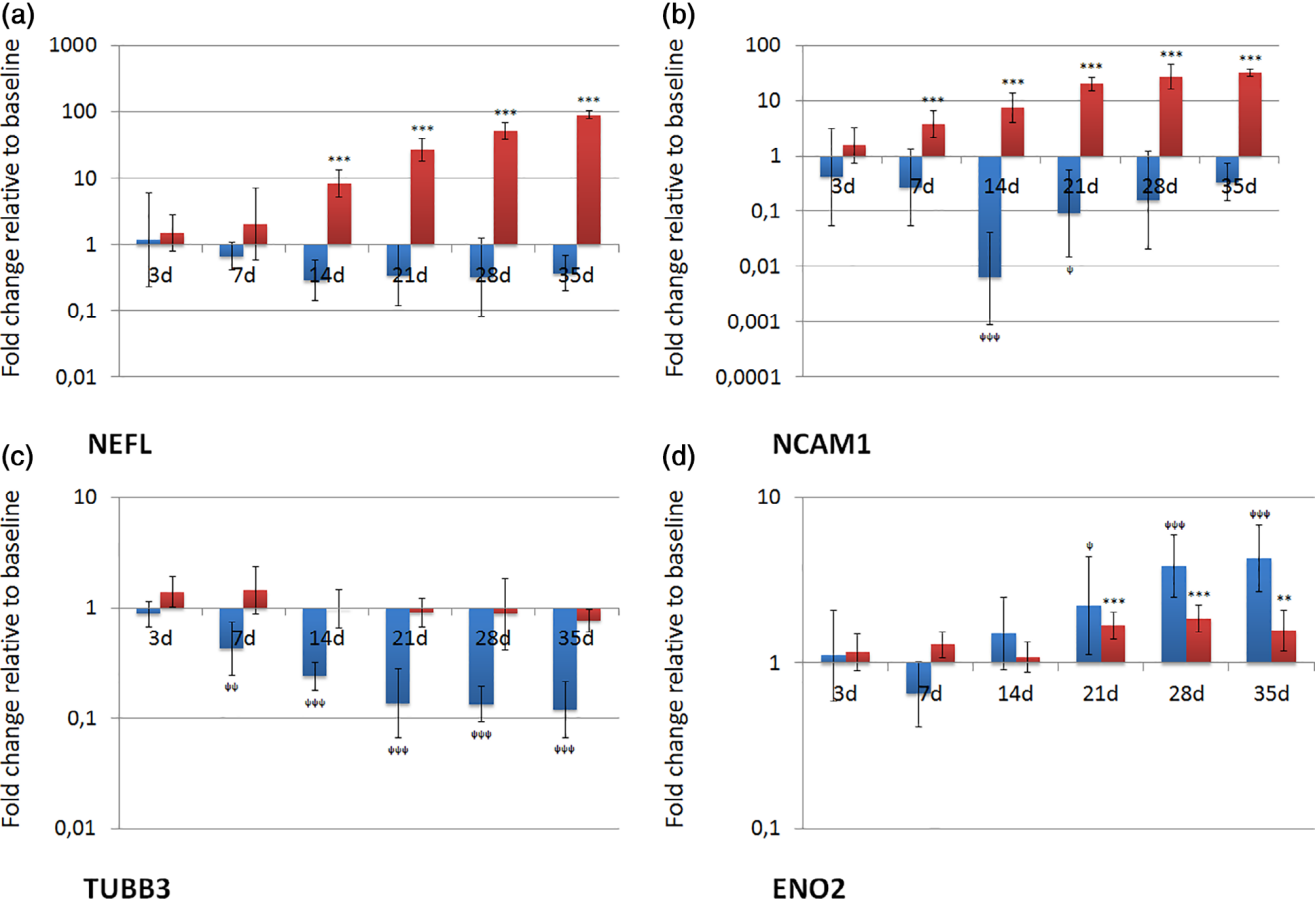
Expression of neuronal markers (NEFL, NCAM1, ENO2 and TUBB3) on transcriptional level during culture in neurogenic differentiation medium (NDM). Fold changes in comparison to baseline are given as recalculated averages with upper and lower confidence interval (cell cultures grown in NDM: red columns; cell cultures grown in CCM: blue columns). Statistically significant differences to the reference value at baseline were identified by one‐way ANOVA with Dunnett's multiple comparison test (*/^φ^ *p* < 0.05; **/^φφ^ *p* < 0.01; ***/^φφφ^ *p* < 0.001)

The observed morphological changes of PIMSCs were further corroborated with gene expression of the neuron‐specific markers NEFL, NCAM1, TUBB3 and ENO2, assessed by qRT‐PCR (Figure 5). PIMSC cultures grown in NDM showed a gradually increasing expression of NEFL with significant values at day 14, 21, 28 and 35 (*p* < 0.001), NCAM1 with significant values at day 7, 14, 21, 28 and 35 (*p* < 0.001), and ENO2 with significant values at day 21, 28 (both *p* < 0.001) and 35 (*p* < 0.01). The expression of TUBB3 did not significantly change under neuro‐inductive culture conditions. In cell cultures grown in CCM, the expression of NEFL did not significantly change, but revealed a trend towards downregulation. The expression of NCAM1 and TUBB3 was significantly downregulated. Statistically significant values were documented at day 14 (*p* < 0.001) and 21 (*p* < 0.05) for NCAM1 and at day 7 (*p* < 0.01), 14, 21, 28 and 35 (*p* < 0.001) for TUBB3. The expression of ENO2 was significantly upregulated at day 21 (*p* < 0.05), 28 and 35 (*p* < 0.001).

### 3.4 Time‐course gene expression of angiogenesis‐related markers

**FIGURE 6:**
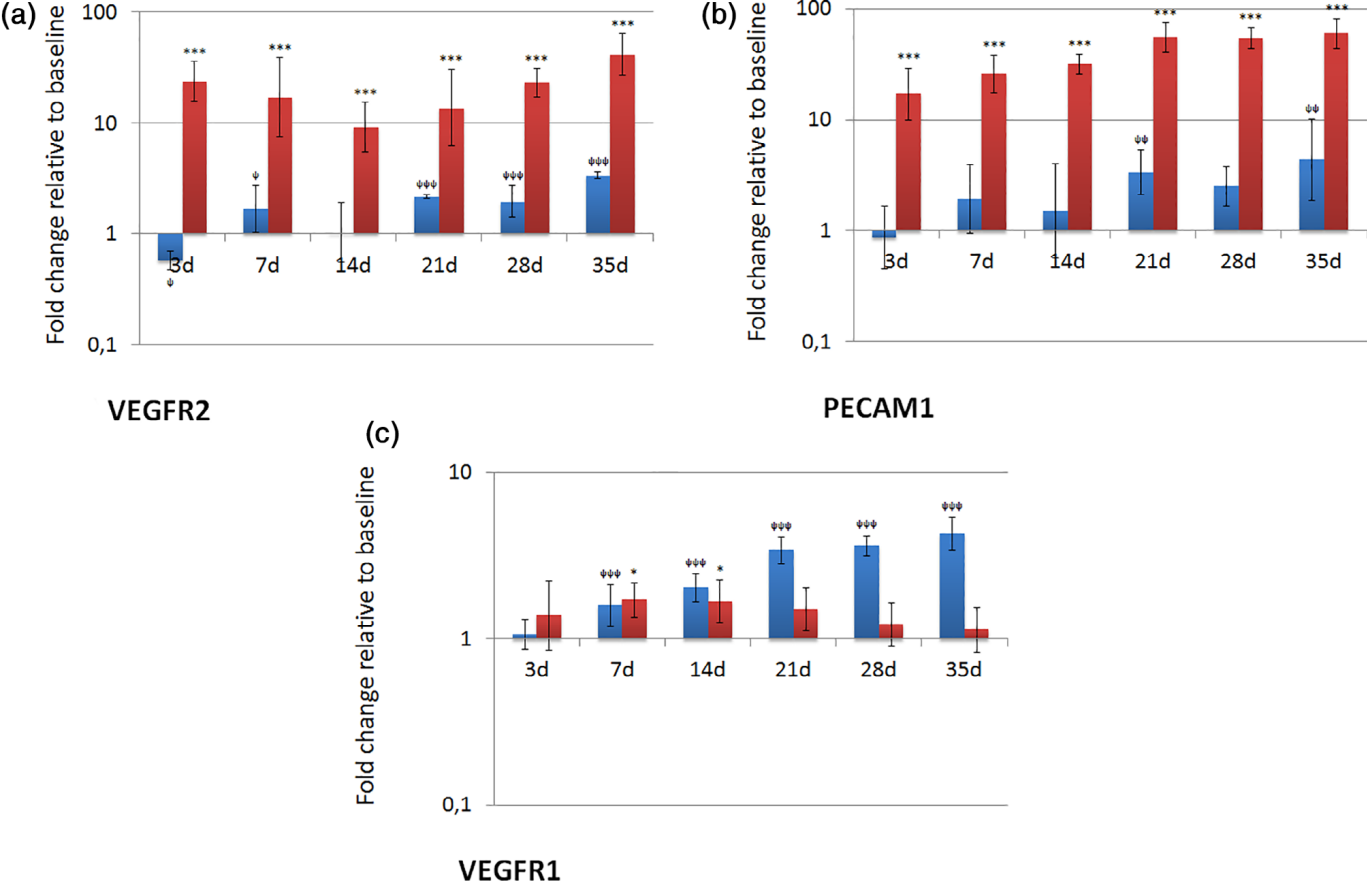
Expression of angiogenic markers (PECAM1, VEGFR1 and VEGFR2) on transcriptional level during culture in angiogenic differentiation medium (ADM). Fold changes in comparison to baseline are given as recalculated averages with upper and lower confidence interval (cell cultures grown in ADM: red columns; cell cultures grown in CCM: blue columns). Statistically significant differences to the reference value at baseline were identified by one‐way ANOVA with Dunnett's multiple comparison test (*/^φ^ *p* < 0.05; **/^φφ^ *p* < 0.01; ***/^φφφ^ *p* < 0.001)

Continuous exposure of PIMSCs to the ADM led to a time‐dependent upregulation of the expression of the angiogenesis‐related markers PECAM1, VEGFR1 and VEGFR2 (Figure 6). The expression of PECAM1 and VEGFR2 was significantly upregulated throughout the entire observation period (*p* < 0.001, respectively). The expression of VEGFR1 showed significant values at day 7 and 14 (*p* < 0.05). The expression of PECAM1 and VEGFR2 was also significantly upregulated in control cultures grown in CCM, but considerably lower and later than in cultures grown in ADM. The expression of VEGFR1 gradually increased during culture in CCM with significant values at day 7, 14, 21, 28 and 35 (*p* < 0.001). This upregulation was clearly higher than in cultures grown in ADM.

### 3.5 Time‐course gene expression of osteogenesis‐related markers

**FIGURE 7:**
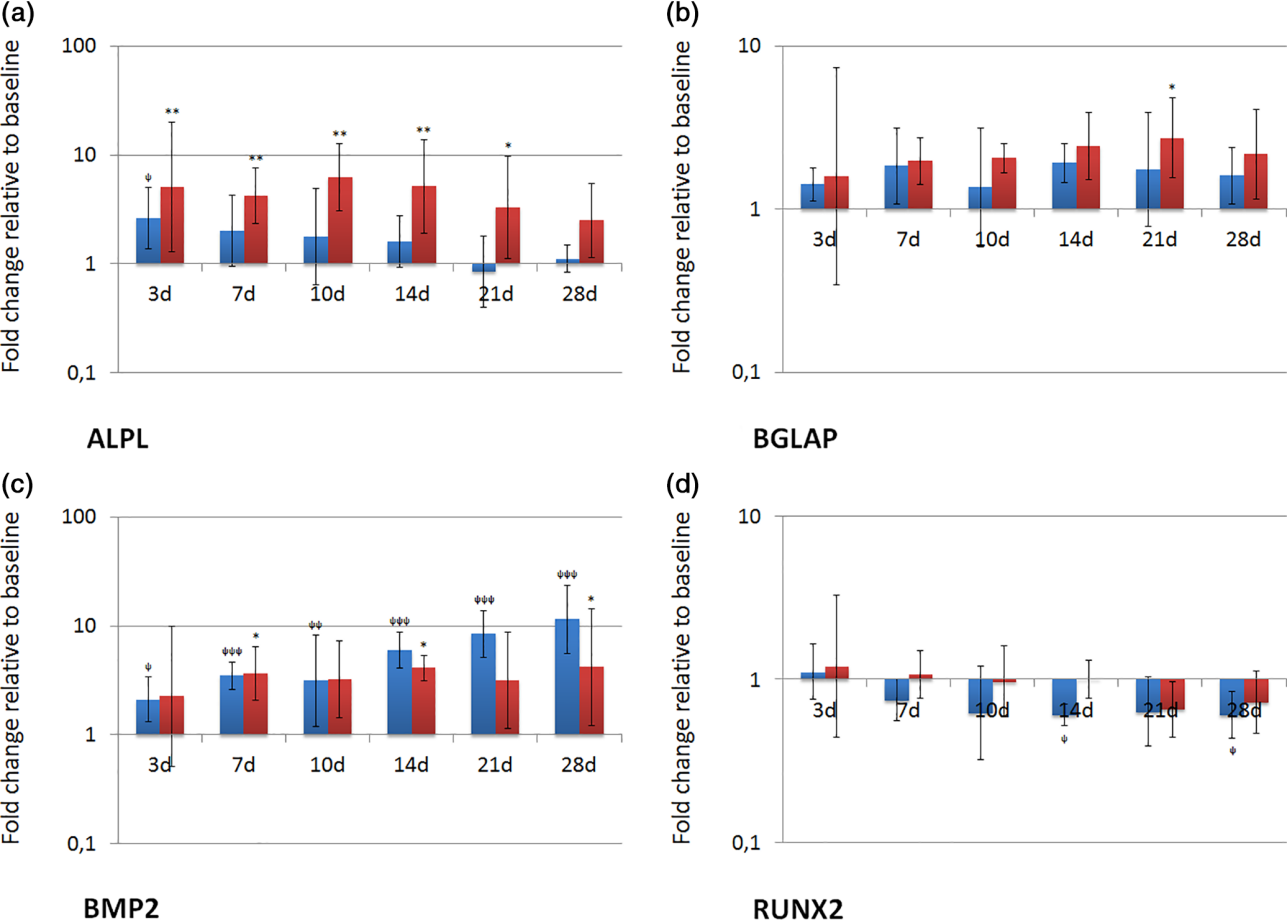
Expression of osteogenic markers (ALPL, BGLAP, BMP2 and RUNX2) on transcriptional level during culture in osteogenic differentiation medium (ODM). Fold changes in comparison to baseline are given as recalculated averages with upper and lower confidence interval (cell cultures grown in ODM: red columns; cell cultures grown in CCM: blue columns). Statistically significant differences to the reference value at baseline were identified by one‐way ANOVA with Dunnett's multiple comparison test (*/^φ^ *p* < 0.05; **/^φφ^ *p* < 0.01; ***/^φφφ^ *p* < 0.001)

The osteogenic differentiation was verified by the expression profiles of the osteogenesis‐related markers ALPL, BGLAP, BMP2 and RUNX2 (Figure 7). In cultures grown in ODM, the expression of ALPL was significantly increased at day 3, 7, 10, 14 (*p* < 0.01, respectively) and 21 (*p* < 0.05), the expression of BGLAP was significantly elevated at day 21 (*p* < 0.05), and the expression of BMP2 was significantly upregulated at day 7, 14 and 28 (*p* < 0.05, respectively). The expression of RUNX2 did not significantly change. Cultivation in CCM led to a significant upregulation of the ALPL (day 3, *p* < 0.05) and BMP2 expression (significant values at day 7, 14, 21 and 28, *p* < 0.001). The expression of BGLAP did not significantly change. The expression of RUNX2 was significantly downregulated at day 14 and 28 (*p* < 0.05).

### 3.6 Mineralized tissue formation by PIMSC cultures

**FIGURE 8:**
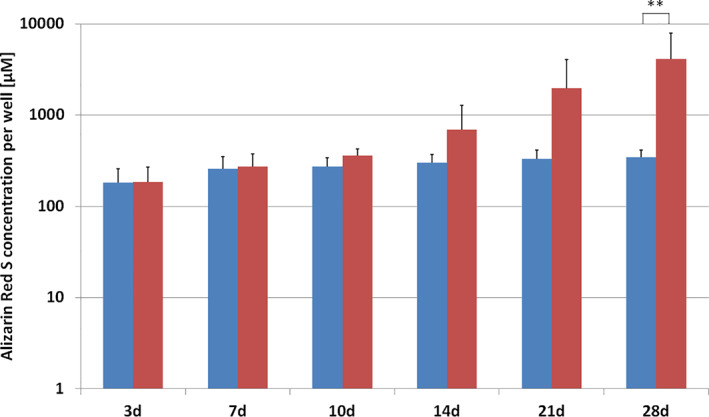
Quantification of mineralized matrix formation using the CPC extraction method. The Alizarin Red S (AR‐S) concentration per well obtained from all donors (*n* = 4) is presented as mean value ± standard deviation (cell cultures grown in ODM: red columns; cell cultures grown in CCM: blue columns). AR‐S concentrations were significantly different at day 28 (one‐way ANOVA with Bonferroni's multiple comparison test; **p < 0.01)

AR‐S positive calcified deposits further confirmed the osteogenic differentiation (Figure 8). During the first 10 days of induction, confluent cells started to produce dense cellular 3D structures in particular at the periphery of the wells. These structures were the first to be mineralized, as shown by the calcium‐specific AR‐S staining. Afterwards, the formation of mineralized tissues progressed towards the center of the well and led to the formation of 3D aggregates, but there were also multiple single mineralized sites throughout the entire adherent monolayer. Matrix mineralization gradually increased and covered almost 100% of the adherent monolayer 3 weeks after the induction of differentiation. However, there were considerable differences with respect to the time‐points when the mineralization started. Thus, mineralized matrix was evident after 14 days in donor 1, after 28 days in donors 2 and 3, and after 21 days in donor 4. This was also confirmed by the spectrophotometric quantification of the mineralized matrix using the CPC extraction method. In cultures grown in ODM, the AR‐S concentration per well continuously increased throughout the entire observation period. A significant difference between cells grown in ODM and CCM was observed after 28 days (*p* < 0.01). The important observation, nonetheless, was that the tissue biopsies of all donors had the potential to form mineralized tissue after induction of differentiation. In non‐induced PIMSC cultures grown in CCM, only negligible matrix mineralization was observed.

## 4 DISCUSSION

PI is associated with progressive loss of supporting bone and impaired healing potential (Park et al., 2015). To our knowledge, this study provides for the first time evidence that cell populations with a regenerative potential exist in the GT of PI lesions despite the presence of an inflammatory infiltrate.

Two of our four tissue donors were smokers. Studies assessing the influence of smoking and nicotine on the properties of MSCs suggest that smoking results in a reduced immunomodulatory capacity, an impaired osteogenic differentiation potential in vitro, and poorer bone regeneration capacity in vivo (Cruz et al., 2019; Sreekumar et al., 2018; Zhao et al., 2018). However, we could not find any difference in the experimental data that would indicate an influence of the smoking behavior of the donor.

The definition of a cell population as MSCs involves the expression of specific epitopes. In the present study, we first performed an extensive immunophenotypic analysis with regard to several stem cell markers (Figures 3 and 4). These immunophenotypic profiles were overall similar in the analyzed PIMSC cultures. The combined expression of both mesenchymal (CD90, CD73, CD105, CD146, STRO1) and embryonic (SSEA4, NANOG, SOX2, OCT4A) stem cell markers is indicative of an enriched stem cell content. In particular, CD90 (99.25% ± 0.36%), CD73 (98.14% ± 1.93%) and CD105 (79.49% ± 27.96%) were strongly expressed by all non‐induced PIMSC cultures. The amount of CD105^+^ cells was significantly lower than that suggested as minimum by the International Society of Cell Therapy (ISCT; Dominici et al., 2006). Accordingly, Tang et al. have shown in a patient‐matched comparative study that the expression of CD105 was significantly lower in cells derived from “inflamed” periodontal sites (61.3% ± 3.5%) when compared to cells derived from healthy periodontal sites (96.2% ± 2.8%; Tang et al., 2016). This suggests that an inflammatory condition has an inhibitory effect on the expression of CD105. An additional analysis was performed for CD146 (75.49% ± 12.87%) and STRO1 (41.30% ± 13.24%). Recently, we investigated the expression of mesenchymal and embryonic stem cell markers in cell cultures established from GT of infrabony periodontal defects (inflamed human periodontal ligament stromal cells—ihPDLSCs; Adam et al., 2020). Overall, the expression profiles of both ihPDLSC and PIMSC cultures showed many similarities. Thus, a high expression of CD73 (97.66% ± 1.92%), CD90 (98.87% ± 0.93%), CD105 (78.02% ± 12.81%) and CD146 (80.64% ± 23.87%) was also observed in ihPDLSC cultures. However, the expression of STRO1 (5.29% ± 3.62%) was significantly lower in ihPDLSC cultures. Contradictory data have also been published concerning the expression of STRO1 in healthy and inflamed periodontal tissues. While some comparative studies observed a higher expression of STRO1 in cells derived from inflamed periodontal sites (Li et al., 2014; Tomasello et al., 2017), others reported a higher expression in cells derived from healthy sites (Park et al., 2011; Tang et al., 2016). The percentage of STRO1^+^ cells ranged from 6.6% to 37.8% in these investigations. The latter is rather consistent with our results.

Another interesting finding of the present study was that PIMSC cultures strongly expressed the embryonic stem cell markers SSEA4 (61.54% ± 19.51%), NANOG (83.43% ± 8.47%), OCT4A (97.94% ± 1.03%) and SOX2 (98.72% ± 0.63%). When we compare these data with those of our recently published study (Adam et al., 2020), it becomes evident that there was a comparable expression of OCT4A (94.60% ± 1.95%) and SOX2 (98.27% ± 0.36%), but a significantly lower expression of SSEA4 (29.76% ± 12.38%) and NANOG (52.09% ± 6.98%) in the ihPDLSC cultures. Several studies have confirmed that cells isolated from healthy periodontal ligament and bone marrow express the embryonic stem cell markers SSEA4, NANOG, OCT4 and SOX2 (Bearden et al., 2017; Kawanabe et al., 2010; Ponnaiyan et al., 2012; Riekstina et al., 2009; Trubiani et al., 2010; Vasandan et al., 2014). However, our data provide evidence that embryonic stem cell markers are also expressed by cells isolated from inflamed peri‐implant and periodontal granulation tissue. In agreement with our results, Tomasello et al. revealed that cell cultures established from periodontally affected teeth have a significantly higher expression of NANOG, OCT4 and SOX2 than cell cultures established from periodontally healthy teeth (Tomasello et al., 2017). Overall, the data of the immunophenotypic analysis suggest that PIMSCs have properties of MSCs.

Our next aim was to investigate, if PIMSC cultures exhibit tri‐lineage differentiation potential. Ivanowski et al. and Donos et al. investigated the biological processes associated with osseointegration of titanium dental implants in humans (Donos, Hamlet, et al., 2011; Donos, Retzepi, et al., 2011; Ivanovski et al., 2011). They reported that genes related to inflammation, angiogenesis, neurogenesis and skeletogenesis were temporally upregulated during the early stages of osseointegration. If neurogenesis, angiogenesis and osteogenesis are required for the osseointegration after implant placement, it appears conclusive that these processes are also required for the regeneration of PI defects. Therefore, our study addressed these three different pathways of differentiation: neurogenesis, angiogenesis and osteogenesis. From a morphological point of view, we observed that PIMSC cultures display heterogeneity. This is not surprising because we used the enzymatic dissociation method to establish PIMSC cultures. This resulted in the release of different cell types with varying size and morphology (Gronthos et al., 2000, 2002). After induction of PIMSC cultures with respective differentiation media, the morphology of PIMSCs turned into characteristic directions (Figure 2). The neurogenic differentiation resulted in the formation of axon‐ and dendrite‐like cell structures. The angiogenic induction led to cells with an endothelial cell‐like morphology arranged in a cobblestone‐like organizational pattern. Finally, the osteogenic differentiation caused colony‐like 3D cell clusters and extensive matrix mineralization. Dense deposits of calcium similar to bone nodules were observed in the cultures following staining with AR‐S, but no sign of mineralization was detected in the control cultures grown in CCM (Figure 2d). In cultures exposed to ODM, the mineralized matrix started to form next to the cellular aggregates and gradually increased until it covered almost 100% of the monolayer at the end of the observation period.

Transcription profiling has broadened the knowledge about the biological processes and signaling pathways associated with the early events of wound healing (Donos, Hamlet, et al., 2011; Donos, Retzepi, et al., 2011). Cells exposed to NDM showed a continuously increasing expression of NEFL, NCAM1 and ENO2 throughout the entire differentiation experiments (Figure 5). The NEFL gene encodes a type IV intermediate filament playing an important role in the intracellular transport of neurotransmitters to axons and dendrites (Leermakers & Zhulina, 2010). NCAM1 encodes a cell adhesion protein, which is involved in cell‐to‐cell and cell‐to‐matrix interactions during development and differentiation (NCAM1 Neural Cell Adhesion Molecule 1, 2020). ENO2 is known to be a useful index of neural maturation and a highly specific marker for neurons and peripheral neuroendocrine cells (Isgrò et al., 2015). Interestingly, the expression of TUBB3 did not significantly change in cells grown in NDM, but significantly decreased in cells grown in CCM. The same expression pattern was observed in ihPDLSC cultures during the neurogenic differentiation experiments (Adam et al., 2019; Adam et al., 2020). As both alveolar bone and periodontal ligament originate from the neural crest, it is not surprising that cells isolated from these tissues exhibit a baseline expression of neural markers (Foudah et al., 2014; Heng et al., 2016).

Continuous exposure of PIMSC cultures to ADM led to a substantial time‐dependent increase in the expression of angiogenesis‐related molecules, including PECAM1 and VEGFR2 (Figure 6). PECAM1 is a member of the immunoglobulin superfamily and likely involved in leukocyte migration, angiogenesis and integrin activation (National Center for Biotechnology Information, 2020). VEGF is a key regulator of angiogenesis (Neufeld et al., 1999). There are two tyrosine kinase receptors (VEGFR1 and VEGFR2) on endothelial cells that bind VEGF with high affinity. VEGF/VEGFR2‐signaling is known to mediate a plethora of cellular functions involved in angiogenesis, like endothelial cell proliferation, migration, and survival (Abhinand et al., 2016; Chappell et al., 2009; Olsson et al., 2006). While the expression of VEGFR2 was gradually increased, the expression of VEGFR1 was markedly suppressed over time. VEGFR1 is assumed to affect vascular development by modulating VEGF‐mediated VEGFR2 signaling (Chappell et al., 2009). The soluble form of VEGFR1 is known to act as a ligand sink that reduces the amount of VEGF available for VEGFR2 binding (Chappell et al., 2009). Accordingly, Shibuya described VEGFR1 as a negative regulator of angiogenesis (Shibuya, 2011).

The ability to regenerate bone is essential for the re‐osseointegration of an implant affected by PI. Therefore, we investigated the expression of several osteogenesis‐related genes during osteoblast differentiation and bone matrix formation induced by dexamethasone, β‐glycerol phosphate and inorganic phosphate. In vivo, the processes of osteoblast differentiation and bone formation are dynamically coordinated by transcription factors (like RUNX2), growth factors (like BMP2) and stage‐specific signal transduction. RUNX2 is expressed by uncommitted mesenchymal cells, upregulated in preosteoblasts and immature osteoblasts, and finally downregulated in mature osteoblasts (Komori, 2010). In our study, the expression of RUNX2 did not significantly change, but exhibited a definite downregulation at day 21 and 28, when pronounced matrix mineralization occurred (Figure 7). At this stage, mature osteoblasts were likely to be present. ALPL is widely used as an early marker of osteoblast differentiation (Yang et al., 2009). In our study, the expression of ALPL was significantly increased within the first 21 days of induction and afterwards downregulated, when matrix mineralization largely covered the culture vessels. This was confirmed by Park et al., who reported a downregulation of ALPL expression during the mineralization process (Park et al., 2009). BGLAP is a non‐collagenous protein associated with the late phase of osteoblast differentiation (Komori, 2010; Viereck et al., 2002). BGLAP is known to act as a regulator of bone mineralization (Neve et al., 2013). Accordingly, we observed a significant upregulation of BGLAP in the advanced stages of the experiments, when distinct matrix mineralization occurred. Bone morphogenic proteins (BMPs) belong to the transforming growth factor β superfamily. Of all BMPs, BMP2 appears to be the most potent inducer of bone formation (Ishikawa et al., 2007). In the present study, BMP2 showed a continuously increasing expression in cells grown in ODM. Interestingly, the expression of BMP2 was even stronger upregulated in cells grown in CCM. In this context, it is important to know that BMPs are not only involved in bone formation, but also in non‐osteogenic developmental processes (Chen et al., 2004).

Taken together, the morphological changes and transcriptional data of our study indicate that PIMSCs have the potential to differentiate into neuron‐, endothelial cell‐, and osteoblast‐like cells. Thus, preservation of GT during regenerative therapy of PI appears to be beneficial for the processes of re‐innervation, re‐vascularization and re‐osseointegration. However, the mutual interaction between MSCs and the local inflammatory microenvironment must also be considered during the healing of PI lesions. There is evidence that an inflammatory microenvironment affects the proliferation, migration/homing, multilineage differentiation potential, and inflammatory cytokine production of MSCs (Fawzy El‐Sayed et al., 2019). Conversely MSCs have been shown to modulate the activity of cells of the innate and adaptive immune system. The immunomodulatory properties of MSCs have mainly been investigated in vitro, but only sparsely in vivo (Zhou et al., 2020). This may be the subject of future animal studies.

Lin et al. investigated, if periodontal ligament stem cells are present in regenerating periodontal tissues (Lin et al., 2008). Molar teeth with class II furcation involvement were surgically treated using guided tissue regeneration and extracted after 6 weeks of healing. The associated regenerating periodontal tissues were assessed using immunohistochemistry, flow cytometry and differentiation assays. The authors reported that putative mesenchymal stromal cells were present in regenerating periodontal defects. The GT used for the present investigation was harvested from PI lesions, where a thorough non‐surgical treatment was performed 3–6 weeks prior to the surgical intervention. The non‐surgical treatment resulted in the resolution of inflammation signs and increased firmness of the peri‐implant soft tissues. Therefore, the GT used in the present study may also be regarded as regenerating peri‐implant tissues.

## 5 CONCLUSION

Cells with MSC‐like properties were isolated from the GT of PI lesions. These cells revealed the capacity to undergo neurogenic, angiogenic and osteogenic differentiation. These findings suggest that inflamed GT of PI lesions have a significant regenerative potential. Further studies considering the immunomodulatory properties are necessary to specify the biological role of PIMSCs for the healing processes of inflamed peri‐implant tissues and their potential application in regenerative treatment strategies.

## CONFLICT OF INTERESTS

The authors declare that they have no competing interests.

## AUTHOR CONTRIBUTIONS

Evangelia Gousopoulou conceived the study, performed the experiments, analyzed and interpreted the data and drafted the manuscript. Athina Bakopoulou participated in the study design, supervised experiments and contributed to drafting of the manuscript. Danae Anastasia Apatzidou and Ingmar Staufenbiel provided the PIMSC samples and critically reviewed the manuscript. Gabriele Leyhausen, Joachim Volk and Werner Geurtsen participated in the experimental design, methodology establishment and critically reviewed the manuscript. Knut Adam conceived the study, supervised and interpreted the experiments, analyzed the data and contributed to drafting of the manuscript. All authors read and approved the final manuscript.

## ETHICS STATEMENT

The present study has been approved by the Institutional Review Board (Ethics Committee of Hannover Medical School, reference number: 1096) and all donors signed an informed consent according to the Declaration of Helsinki.

## Data Availability

The datasets generated and/or analysed during the current study are not publicly available but are available from the corresponding author on reasonable request.
